# SmallTalk: a novel small‐sized fusion tag for peptide expression and purification

**DOI:** 10.1002/2211-5463.70147

**Published:** 2025-11-11

**Authors:** Atika Tariq, Nestor G. Casillas‐Vega, Alma Gomez‐Loredo, Xristo Zarate

**Affiliations:** ^1^ Facultad de Ciencias Quimicas Universidad Autonoma de Nuevo Leon San Nicolas de los Garza NL Mexico; ^2^ Departamento de Patologia Clinica, Hospital Universitario Dr. Jose Eleuterio Gonzalez, Facultad de Medicina Universidad Autonoma de Nuevo Leon Monterrey NL Mexico; ^3^ Facultad de Ciencias Quimicas, Centro de Investigacion en Biotecnologia y Nanotecnologia, Parque de Investigacion e Innovacion Tecnologica Universidad Autonoma de Nuevo Leon Apodaca NL Mexico

**Keywords:** antimicrobial peptides, Bin1b, *Escherichia coli*, IMAC, recombinant peptide expression and purification, SmallTalk, SmbP

## Abstract

Recombinant protein production in *Escherichia coli* is a fundamental aspect of biotechnology. Fusion tags are commonly used to enhance solubility and facilitate purification. However, these tags can lead to challenges such as low yields, complicated purification processes, and the necessity for tag removal, especially when dealing with peptides. This study introduces a novel fusion tag called SmallTalk, a truncated version of the small metal‐binding protein SmbP. Weighing in at 5 kDa, SmallTalk includes two of the four α‐helices found in SmbP. It retains the ability to bind Ni(II) ions, which enables purification through IMAC. In this work, we assessed the efficiency of SmallTalk in expressing and purifying both a model protein, the green fluorescent protein, and the antimicrobial peptide Bin1b. Both proteins were effectively expressed and purified using IMAC, demonstrating SmallTalk's value as an affinity tag, yielding 7.2 mg·L^−1^ of cell culture for the green fluorescent protein and up to 9.8 mg·L^−1^ for Bin1b. Antimicrobial assays conducted with SmallTalk‐tagged Bin1b showed activity against *Staphylococcus aureus*, *Escherichia coli*, *Klebsiella pneumoniae*, and *Pseudomonas aeruginosa*, with minimum inhibitory concentrations ranging from 7.5 to 22.5 μm. Importantly, SmallTalk enabled the full retention of Bin1b's antimicrobial activity without the need for its removal, significantly simplifying the production process. These findings indicate that SmallTalk provides a promising strategy for the recombinant production of peptides. This tag has the potential to enhance the expression, purification, and functional analysis of antimicrobial peptides, which are increasingly being pursued as alternatives to antibiotics in the fight against antimicrobial resistance.

## Graphical abstract

The SmallTalk fusion tag allows for the efficient expression and purification of soluble recombinant proteins or peptides in *Escherichia coli*. Testing with SmallTalk‐GFP confirmed that the proteins were soluble and folded correctly, while SmallTalk‐Bin1b maintained its antimicrobial activity against various bacterial isolates. This streamlined workflow showcases the versatility of the SmallTalk system in producing functional, biologically active proteins and peptides.

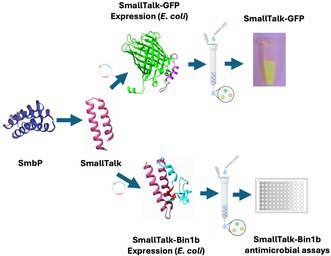

**Abbreviations**
AMPs: antimicrobial peptides
GST: glutathione S‐transferase
IMAC: immobilized‐metal affinity chromatography
IPTG: Isopropyl β‐d‐1‐thiogalactopyranoside
LB: Luria‐Bertani broth
OD_600_
: optical density at 600 nm
SDS/PAGE: sodium dodecyl sulfate polyacrylamide gel electrophoresis
Tris: Tris(hydroxymethyl)‐aminomethane

*Escherichia coli* is still the first choice for recombinant protein production, favored for its simplicity, low cost, and scalability [1]. One of its most valuable attributes is its ability to produce fusion proteins, allowing scientists to obtain pure proteins in higher concentrations with few purification steps [2]. Fusion proteins are crucial in recombinant technology, as they facilitate solubility, folding, and purification [3]. However, the efficiency of any fusion protein tag varies significantly depending on the nature of the target protein [4]. This variability highlights the need for a diversity of fusion tags, each optimized for specific protein or peptide characteristics. Despite the advantages of conventional fusion tags such as glutathione‐S‐transferase (GST, 26 kDa) and maltose‐binding protein (MBP, 42 kDa), challenges such as low final yields, formation of inclusion bodies, and complex tag removal steps persist [5, 6]. These limitations have encouraged the search for smaller, more efficient fusion tags to overcome these obstacles, especially when producing recombinant peptides.

Recent advances in structural and synthetic biology have paved the way for discovering and designing novel proteins with potential commercial and therapeutic applications [7]. This has intensified the need for effective fusion tags expressing these newly discovered proteins in functional quantities in *E. coli*. Fusion tags are generally classified as solubility enhancers or affinity tags; however, a select few can function as both [8]. Considering the exponential growth of protein therapeutics, there is a critical need for fusion tags that streamline expression and purification while maintaining the bioactivity of the target protein [9].

The small metal‐binding protein SmbP, a 9.9 kDa polypeptide isolated from the periplasm of *Nitrosomonas europaea*, has been a promising candidate. SmbP's ability to bind divalent metal ions, such as Cu(II) and Ni(II), makes it an ideal tag for immobilized metal affinity chromatography (IMAC) [10]. Its low molecular weight, significantly smaller than traditional fusion tags such as GST and MBP, reduces the burden on cellular machinery, increasing protein yield [11]. Moreover, the small size of SmbP minimizes the loss of protein yield during tag removal, which is particularly beneficial when producing peptides [12].

SmbP has been employed in the recombinant production of various therapeutic proteins and peptides, including the human growth hormone [13], the antimicrobial peptide LL‐37 [14], the new hybrid antimicrobial peptide LL‐37_Renalexin [15], the anionic antimicrobial peptide scygonadin [16], and defensins such as VpDef and Bin1b [17, 18]. Building on these accomplishments, we have engineered a new fusion protein tag, which we have named ‘SmallTalk’.

SmallTalk is a truncated SmbP version containing only two of its four α‐helices, resulting in a molecular weight of just 5 kDa. This reduced size makes SmallTalk particularly suitable for expressing and purifying peptides, such as antimicrobial peptides (AMPs), hopefully without compromising their biological activity.

AMPs have garnered significant attention as alternatives to traditional antibiotics, given their broad‐spectrum activity and efficacy against drug‐resistant pathogens [19]. The increasing prevalence of antimicrobial resistance has accelerated interest in small peptide drugs, which now represent a rapidly growing class of therapeutic candidates [20]. These peptides, often produced in limited quantities, require efficient expression systems with fusion tags that optimize yield and ease of purification while maintaining bioactivity [7]. SmallTalk, with its potential to streamline the production of antimicrobial peptides, offers a forward‐looking and optimistic perspective in this context.

SmallTalk was designed with these requirements in mind. Our preliminary studies suggest that SmallTalk retains most of the beneficial attributes of SmbP, including efficient expression and purification via IMAC. More importantly, SmallTalk allows the attached peptide to exhibit total antimicrobial activity without requiring tag removal. This feature represents a significant advancement in peptide production, as it eliminates the need for enzymatic cleavage, an often expensive and time‐consuming process, thereby facilitating functional analysis on a laboratory scale.

In this study, we tested the performance of SmallTalk using two models: the standard green fluorescent protein (GFP) and the antimicrobial peptide Bin1b. Both proteins were expressed in *E. coli* and purified using IMAC, confirming the affinity capabilities of SmallTalk. Antimicrobial assays using SmallTalk‐Bin1b demonstrated robust activity against Gram‐positive and Gram‐negative bacterial strains, with minimum inhibitory concentrations (MICs) comparable to or lower than those observed for Bin1b produced with other fusion tags.

## Materials and methods

### Sequence identification and *in‐silico* structure validation of SmallTalk

The amino sequence of SmbP and its 3D structure in PDB format was retrieved from RCSB PDB. The accession ID of SmbP from *Nitrosomonas europaea* in PDB is 3U8V. The confirmation of SmbP amino acid sequence using the blast tool on the NCBI website, with the accession number of SmbP in NCBI being WP_011112924.1 and PDB‐determined 3D structure of SmbP is also linked with the said accession numbers. The nickel affinity of SmbP, attributed to PubChem compound ID of Nickel, is the apparent specification of this protein. The structure of SmbP, consisting of four alpha helices, was aligned using ucsf chimera. The aim was to select the amino acid sequence referring to the 3rd and 4th alpha helices of the SmbP correctly, so as not to disrupt its conformation. Structure modeling of SmallTalk from its putative amino acid sequence was done using two different methods of structure prediction: homology modeling using Swiss Modeler and threading using i‐tasser. The resulting structure was analyzed using the qmean value and Ramachandran plot. Ramachandran plots were generated using PDBsum (https://www.ebi.ac.uk/thornton‐srv/databases/pdbsum/). The structure of SmallTalk‐Bin1b was predicted using i‐tasser as well, which is a homology‐independent tool, and the model was generated using the iterative threading approach.

### DNA constructs, SmallTalk‐GFP and SmallTalk‐Bin1b

Primers were designed to amplify the designated region of SmallTalk through PCR amplification using SmbP DNA as a template. The restriction site of NdeI was added to the flanking side of the forward primer, and the KpnI restriction site was added to the flank of the reverse primer. These restriction sites have been chosen to clone the SmallTalk DNA fragment into a pET‐30a(+) (EMD Millipore, Darmstadt, Germany) parent plasmid that already contains GFP‐tagged SmbP, flanked by the same restriction sites. The amplification of SmallTalk was performed using standard PCR with ThermoFisher (Waltham, MA, USA) *Taq* DNA polymerase. PCR cycle steps deployed for the thermal cycler are as follows: initial denaturation for 1 min at 95 °C, denaturation at 95 °C for 30 s, primer annealing at 55 °C for 45 s, elongation at 72 °C for 1 min, and final elongation at 72 °C for 5 min, with 30 reaction cycles of amplification. The amplified product was visualized under UV using a 1.5% agarose gel stained with ethidium bromide. The amplification product was purified with the MEGAquick‐spin Plus Total Fragment DNA Purification Kit. pET‐30a_SmbP‐GFP, already prepared in a previous laboratory study, was digested with NdeI and KpnI restriction enzymes, following the manufacturer's protocol (New England Biolabs, Ipswich, MA, USA). The same reaction was performed with the purified SmallTalk amplicon. Linearized pET‐30a(+) backbone DNA and SmallTalk DNA were purified using a MEGAquick‐Spin Gel Extraction Kit. The ligation reaction mixture of pET‐30a(+) plasmid DNA (50 ng) and SmallTalk (18 ng) following a 1 : 10 vector to insert ratio was carried out following the manufacturer's protocol (New England Biolabs, T4 DNA ligase). 5 μL of the ligation mixture was transformed for propagation into chemically competent *E. coli* DH5α cells using the heat shock method (42 °C for 45 s). After plating on LB/kanamycin (30 μg·mL^−1^), cells were incubated at 37 °C overnight. Transformant cells were screened for positive clones. Colonies were picked and grown in 2 mL of LB/kanamycin broth for 16 h. Cells were harvested, and plasmid DNA isolation was performed using the iNtRON DNA‐Spin Plasmid DNA Purification Kit. The presence of the gene was confirmed by PCR amplification using the T7 forward and reverse primers. Confirmed plasmid constructs were sequenced by STARSEQ (Instituto de Biotecnologia, UNAM, Mexico), and results were analyzed using bioedit (version 7.7) software. Pairwise alignment of the returned sequence and theoretically curated nucleotide sequence of the SmallTalk‐GFP construct was conducted using Emboss Needle (https://www.ebi.ac.uk/jdispatcher/psa/emboss_needle).

A 118‐nucleotide sequence encoding SmallTalk‐Bin1b (Bin1b accession number in Antimicrobial Peptide Databases = AP01592) optimized according to the codon usage preferences in *E. coli* was synthesized by GenScript (Piscataway, NJ, USA) and cloned into pUC57. The protein‐coding DNA region is flanked by NdeI and XhoI restriction sites. Both pET‐30a(+) and pUC57 cloned with SmallTalk‐Bin1b were digested with NdeI and XhoI restriction enzymes. Restricted fragments were loaded on a 1% agarose gel stained with ethidium bromide. The target DNA fragments were purified using a MEGAquick‐Spin DNA Purification Kit and then ligated with T4 DNA ligase. The ligation mix was transformed into DH5α, and DNA from positive colonies was purified and further confirmed for the presence of the designed plasmid DNA molecule through PCR amplification of the T7 region, followed by Sanger sequencing, as mentioned above. For antimicrobial assays comparison with SmallTalk‐Bin1b, the SmbP‐Bin1b DNA construct was synthesized and cloned into pET‐30a(+) by GenScript. It was transformed into SHuffle T7(DE3) for small‐scale expression and one‐liter expression alongside SmallTalk‐Bin1b.

### Protein expression and purification

The confirmed DNA construct for SmallTalk‐GFP was transformed into *E. coli* BL21(DE3); SmallTalk‐Bin1b and SmbP‐Bin1b were transformed into SHuffle T7(DE3) cells (New England Biolabs). Single colonies were picked and used to inoculate 2 mL of LB‐kanamycin broth. After 16 h of incubation at 37 °C with shaking at 220 r.p.m., 10 μL from this overnight‐grown culture was used to inoculate 10 mL of LB‐Kanamycin in small flasks. The bacterial culture was grown at 37 °C and a shaking speed of 220 r.p.m. until the optical density at 600 nm (OD_600_) reached 0.4–0.6. At this point, the flasks were allowed to cool to room temperature, and isopropyl β‐d‐1‐thiogalactopyranoside (IPTG) was added to a final concentration of 0.1 mm to induce the expression of recombinant proteins. Expression was performed at varying time values (4 and 16 h) and temperatures (37 °C and 25 °C) to determine the optimal conditions. Cells were harvested by centrifugation at 16 250 ***g*** for 5 min in a 2‐mL Eppendorf tube. After decanting the supernatant, 120 μL of lysis buffer 1 (50 mm Tris–HCl, pH 8.0) was added, along with 150 μL of glass beads, and the mixture was vortexed for 5 min. After removing the glass beads, the mixture was centrifuged at 16 250 ***g*** for 5 min; the supernatant was taken into a new tube. Sample buffer 6× (4 mL of glycerol, 2.4 mL of 1 m Tris–HCl buffer pH 6.8, 0.8 g of SDS, 4 mg of bromophenol blue, 0.5 mL of beta‐mercaptoethanol, and 3.1 mL of sterilized distilled water, total volume of 10 mL) was added to the supernatant at a final concentration of 1×. The mix was boiled for 10 min and samples were analyzed using standard SDS/PAGE procedures. Polyacrylamide gels for SDS/PAGE of small‐scale expression analysis were prepared using a 40% (w/v) acrylamide:bisacrylamide (29 : 1 ratio) solution (BIO BASIC, Amherst, NY, USA). The resolving gel (12%) was prepared by mixing the following components: 1.875 mL of 40% acrylamide solution, 1.25 mL of 1.5 m Tris–HCl buffer (pH 8.8), 100 μL of 10% SDS, 1.825 mL of distilled water, 100 μL of freshly prepared 10% ammonium persulfate (APS; Sigma‐Aldrich), and 10 μL of TEMED (Sigma‐Aldrich, Darmstadt, Germany). For the stacking gel (4%), the following were combined: 0.25 mL of 40% acrylamide solution (29 : 1), 0.62 mL of 0.5 m Tris–HCl buffer (pH 6.8), 50 μL of 10% SDS, 1.6 mL of distilled water, 50 μL of 10% APS, and 5 μL of TEMED.

Gels were run in 1× standard Tris‐Glycine SDS running buffer, prepared from a 10× stock solution that contains 0.25 m Tris base, 1.92 m Glycine, and 1% SDS, using a mini‐vertical electrophoresis unit (Mini‐PROTEAN system; Bio‐Rad Laboratories, Hercules, CA, USA). For the molecular weight estimation of small‐scale expressions of SmallTalk‐GFP, an unstained protein standard with a broad range (10–200 kDa, New England Biolabs) was used, as shown in Fig. 4. In contrast, a wide‐range two‐color protein standard by BIO BASIC (10–250 kDa) is used in Fig. 5 for SmallTalk‐Bin1b. After electrophoresis, the gels were stained with staining solution (0.1% Coomassie Brilliant Blue R‐250, 50% methanol, 10% glacial acetic acid) and then destained by washing twice with distilled water and destaining solution (40% methanol, 10% glacial acetic acid) until the bands became visible.

To prepare for large‐scale expression intended for protein purification, 1 L of LB media was prepared and distributed into eight 500‐mL baffled flasks (125 mL per flask). Before inoculating each flask with the overnight culture, kanamycin was added to reach a final concentration of 30 μg·mL^−1^, and the flasks were inoculated with 125 μL of the overnight culture. The cells were allowed to grow at 37 °C with shaking at 220 r.p.m. until they reached an OD_600_ of 0.4–0.6. Recombinant protein expression was induced with IPTG at a final concentration of 0.1 mm once the flasks cooled to room temperature. The cultures were then maintained under previously optimized conditions (25 °C at 220 r.p.m. for 16 h). Cells were harvested in 50‐mL Falcon tubes by centrifugation at 17 000 ***g*** for 15 min in a refrigerated centrifuge. Cell pellets harboring the recombinant protein expression were resuspended in 30 mL of cold lysis buffer (50 mm Tris–HCl, 500 mm NaCl, pH 8.0) with 30 mL of 0.1 mm glass beads. After resuspension of the cell pellet and before vortexing, antifoam 204 (Sigma‐Aldrich) and protease inhibitor cocktail without EDTA (Sigma‐Aldrich) were added. Cells were lysed by vortexing for 10 min while maintaining the refrigerated conditions. Once the lysis was complete, the Falcon tubes were centrifuged at 8500 ***g*** for 15 min at 4 °C, resulting in a pellet of cell debris and a clear soluble cell lysate. Recombinant proteins (GFP and Bin1b) tagged with SmallTalk were purified individually using IMAC and the ÄKTA Prime Plus System (GE HealthCare, Chicago, IL, USA). A 1‐mL HisTrap FF column charged with Ni(II) was used for purification. The column was first equilibrated with 5 column volumes (CVs) of lysis buffer: 50 mm Tris–HCl, 500 mm NaCl, pH 8. To avoid overpressure error, the clarified whole‐cell lysate was loaded onto the column under persistent conditions of 0.5 mL·min^−1^ flow rate and 0.5 MPa pressure. The column was then washed with 3 CVs of wash buffer (50 mm Tris–HCl, 500 mm NaCl, 2.5 mm imidazole, pH 8) until a steady baseline was achieved. Elution of the recombinant protein was performed using an elution buffer composed of 50 mm Tris–HCl, 500 mm NaCl, 200 mm imidazole, pH 8. Elution fractions, the flow‐through, and the cell lysate were analyzed using 12% or 15% SDS/PAGE to visualize the presence of the target recombinant proteins. SmallTalk‐Bin1b underwent additional purification through anion exchange chromatography. After affinity chromatography, the pooled fractions were dialyzed against a 50 mm Tris–HCl buffer pH 8 and loaded onto a 1 mL HiTrap Q FF column equilibrated with the same buffer. The column was washed once the sample was fully loaded until no further protein absorption was observed. SmallTalk‐Bin1b was eluted by applying a NaCl gradient up to 1 m. Purified protein samples from 1‐L experiments were analyzed using the TGX Stain‐Free™ FastCast™ Acrylamide Kit, 12%, and visualized in BioRad's ChemiDoc™ MP Imaging System. The size marker used for these experiments was Precision Plus Protein™ All Blue Prestained Protein Standards as shown in Figs 6 and 7. The purity of the bands was quantified using imagej software, provided by the National Institutes of Health (NIH) [21, 22].

### Bioreactor‐based production of antimicrobial compound

The production of SmallTalk‐Bin1b was also carried out in a controlled bioreactor environment, with continuous monitoring of critical parameters to ensure optimal microbial growth and recombinant peptide production. The bioreactor experiment was conducted in an Auxo V1 Quad 1 L Bioreactor System (SIMATIC HMI‐controlled) for a total duration of approximately 20 h, with a three‐hour growth period initiated by inoculating with 2% v/v of an overnight‐grown inoculum. Kanamycin (50 μg·mL^−1^) was added at the time of inoculation to maintain plasmid selection, and Antifoam 204 (0.1% v/v) was introduced to prevent excessive foaming during aeration and agitation in the bioreactor. The initial temperature was set at 37 °C for the growth phase until OD_600_ reached a value of 0.6–0.8; then the culture was induced with IPTG to a final concentration of 1 mm, and the temperature was reduced to 25 °C for 16 h to facilitate proper folding and minimize inclusion body formation. Throughout the experiment, key parameters were monitored and maintained to ensure optimal conditions for antimicrobial production. The pH (Process Value) ranged from the upper limit of 6.46 to the lower limit of 8.81, with a set point of 7.1. To maintain pH during *E. coli* growth, since the cells naturally acidify the medium through metabolic by‐products, the culture pH was precisely controlled via the automated addition of ammonium hydroxide (NH₄OH) with a set point (SP) of 7.1, maintained throughout the entire experimental duration. The DO (Dissolved Oxygen) levels stabilized at 96.68%, suggesting efficient aeration. Meanwhile, temperature control remained within operational limits, and agitation was maintained to support mixing and oxygen transfer. Cell harvest, cell lysis, and protein purification from the soluble cell lysate were carried out using the same methods mentioned earlier in Section “Protein expression and purification” for 1 L flask‐based expression.

### Antimicrobial activity of SmallTalk‐Bin1b

The MIC of SmallTalk‐Bin1b was evaluated using a dose–response assay. The antimicrobial activity was tested against the Gram‐positive bacterium *Staphylococcus aureus* (ATCC 25923) as well as several Gram‐negative strains, including *Escherichia coli* (ATCC 25922), *Klebsiella pneumoniae*, and *Pseudomonas aeruginosa*. Additionally, MIC assays were conducted for SmbP‐Bin1b against *S. aureus* and *E. coli* to compare its effectiveness with that of SmallTalk‐Bin1b. The clinical isolates of *K. pneumoniae* and *P. aeruginosa* used in these tests were provided by the University Hospital. The MIC assays were performed according to the guidelines set by the National Committee for Clinical Laboratory Standards (NCCLS), using the broth microdilution method. Single colonies of the bacterial strains were picked to inoculate 5 mL of Tryptone Soy Broth (TSB), which is composed of 17.0 g tryptone, 3.0 g soy peptone, 2.5 g glucose, 5.0 g sodium chloride, and 2.5 g dipotassium phosphate (pH 7.3). The inoculated broth was incubated overnight at 37 °C with shaking at 220 r.p.m. On the following day, 5 μL of the overnight culture was used to inoculate 5 mL of TSB and was incubated with shaking at 37 °C until the optical density reached 0.8–1.0, indicating the mid‐log growth phase. The cultures were then serially diluted in 1× PBS buffer to achieve a concentration of 1 × 10^5^ CFU·mL^−1^. A 96‐well microtiter plate was utilized to conduct the twofold broth microdilution assay, with a total assay volume of 200 μL in each well. Each well contained 100 μL of the diluted antimicrobial peptide (with concentrations ranging from 30 to 0.5 μm in 1× PBS buffer), 80 μL of the bacterial suspension, and 20 μL of TSB to support bacterial growth. The contents of each well were gently mixed with a pipette tip to avoid cross‐contamination. A negative control, consisting of a bacterial suspension and 1× PBS buffer, was included in the experimental design. The plate was incubated for 16 h at 37 °C with shaking. Optical density at 600 nm was measured immediately before and after incubation; preincubation OD values were subtracted from postincubation values to correct for background [17, 18]. MIC is defined as the concentration of the antimicrobial agent that prevents visible turbidity and bacterial growth compared to the negative control [23]. Each MIC assay was repeated 2–3 times independently to confirm reproducibility. Once consistent results were obtained, one representative experiment was selected for complete statistical analysis. Within this experiment, each condition was tested in triplicate wells (*n* = 3). Results were normalized to the untreated negative control, which was defined as 100% growth. Data are presented as mean ± standard deviation of triplicates. Statistical comparisons between treatments and the negative control were carried out using Student's *t*‐tests.

## Results and Discussion

### *In silico* structure prediction and analysis

Since the sequence of SmallTalk is extracted from SmbP, the homology modeling method of structure prediction was chosen. Homology modeling is the most accurate method of structure prediction when a template with significant sequence similarity already exists [24]. Swiss Model is a homology modeling‐based web server that searches for the most suitable template based on blast and HHblits search algorithms [25]. From the list of templates returned by the Swiss Model, the crystallographic structure of SmbP (PDB Id 3u8v.1) was selected to build the model. Its QMEANDisCo and GMQE values qualify a predicted model. The QMEANDisCo global score is an advanced version of the QMEAN score with values ranging from 0 to 1, where higher values represent accuracy. The predicted model's QMEANDisCo is 0.7, while the Global Model Quality Estimate (GMQE) is 0.71, representing the model's reliability.

**Fig. 1:**
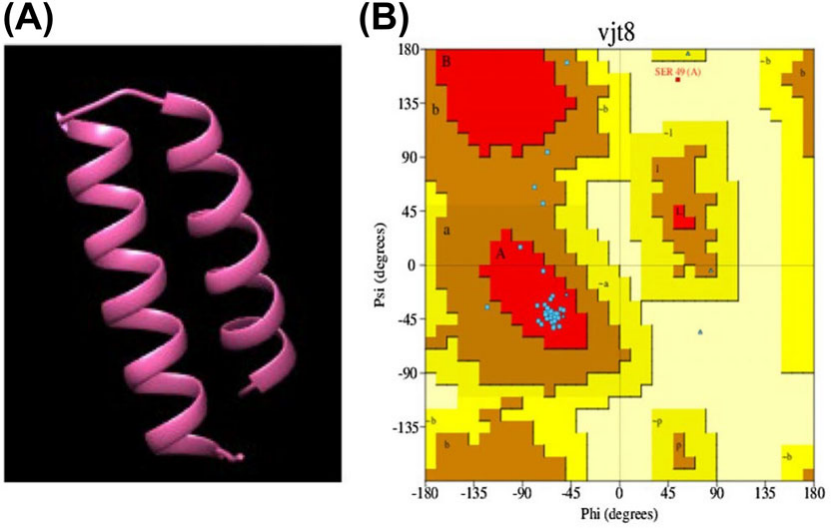
Structure prediction and analysis of SmallTalk. (A) Predicted model of SmallTalk using SmbP as lead structure generated through the i‐tasser server. (B) Ramachandran plot generated using PDBsum of the predicted model of SmallTalk, with 95% of residues in the favorable region, depicting the conformational confidence of the newly developed protein tag.

The predicted structure of SmallTalk using i‐tasser is depicted in Fig. 1A; the Ramachandran plot (Fig. 1B) shows that 95.35% of residues are in a favored region where the acceptable range starts from 85%. Further, the values of Ramachandran outliers 0.00%, molprobity Score 1.20, Clash score 0.00, bad bonds 0/343, and bad angles 10/460 showed the model to be a proper fit [26, 27]. As Ramachandran's plot indicates the prediction of structural stereochemical properties of new molecules, the plot of SmallTalk depicts that 95.35% of residues fall in favorable regions. It shows that the conformation is energetically favorable and consistent with the allowed structures of proteins [26]. A molprobity score of 1.20 indicates that the model is of very good quality and bond angles and lengths are close to ideal values with minimum steric clashes. The C‐beta deviation value is a measure that shows the displacement of the C‐beta atom from its ideal geometry. Our predicted structure returned a zero value of C‐beta, which means C‐beta atoms are positioned exactly as expected, provided C‐beta is a key structural component of an amino acid. All molprobity results are summarized in Table 1.

**Table 1:** molprobity results for SmallTalk.

molprobity score	1.20
Clash score	0.00
Ramachandran favored	95.35%
Ramachandran outliers	0.00%
Rotamer outliers (A8 HIS)	3.12%
C‐beta deviations	0
Bad bonds	0/343
Bad angels A34 HIS, A15 HIS, A22 HIS, A30 HIS, A41 HIS, A8 HIS, A27 HIS, A11 HIS	10/460

**Fig. 2:**
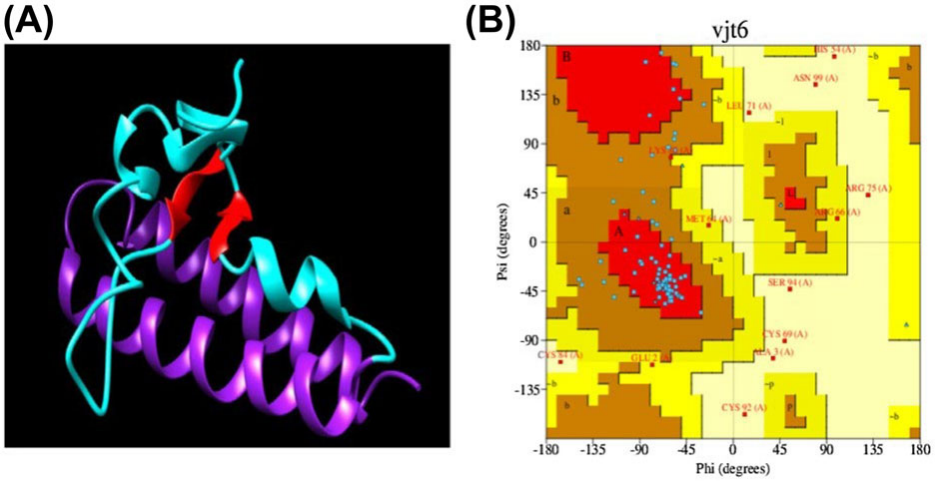
Structure prediction and analysis of SmallTalk‐Bin1b. (A) Predicted model of SmallTalk‐Bin1b generated using the i‐tasser server; alpha helices of SmallTalk are depicted in purple color, cyan colored structures with embedded red colored beta sheets represent Bin1b. (B) Ramachandran plot generated using PDBsum of the predicted model of SmallTalk‐Bin1b.

The predicted structure of SmallTalk‐Bin1b is shown in Fig. 2A. The Ramachandran plot of the predicted model of SmallTalk‐Bin1b generated using PDBSum is shown in Fig. 2B. The plot results showed that 88% of residues fall in favorable regions (60% most favorable, 24% additional, and 10% generously allowed regions), while 10% fall in disallowed regions. The high percentage of disallowed residues and residues in additional and generously favorable regions compared to most favorable regions can be explained by the presence of 12 glycine residues in a 100 amino acid structure. High glycine increases the conformational freedom and overall flexibility of protein structure.

The physicochemical properties of SmallTalk were calculated using ProtParam (http://www.expasy.org/tools/proparameter). The solubility of SmallTalk was calculated using Protein‐Sol (https://protein‐sol.manchester.ac.uk/), which resulted in the solubility index of SmallTalk being 0.89, where the average of an experimental dataset representing the average soluble protein from *E. coli* is 0.45 and values above this depict higher solubility. The pI of SmallTalk also gives insight into the hydrophilic nature of the tag and thus coincides with estimated solubility index values (Table 2).

**Table 2:** Physicochemical parameters for SmallTalk using ProtParam.

Amino acid sequence	SEAGGNTHVGHGIKHLEDAIKHGEEGHVGHATKHAQEAIEHLRASEHKSH
Number of amino acids	50
Molecular weight	5372.78 da
Theoretical pI	6.33
Aliphatic index	62.60
Grand average of hydropathicity (GRAVY)	−1.068
The instability index (II)	31.01 (this classifies the protein as stable)

SmallTalk's design, retaining only two of SmbP's alpha helices, maintains a high level of structural stability. This supports the hypothesis that the smaller fusion tag can still perform critical roles, such as solubilization and binding, making it potentially valuable for protein purification schemes. Notably, the physicochemical properties of SmallTalk further demonstrate its utility, with a high solubility index (0.89) and a pI that contributes to its hydrophilicity, aiding in its potential role as a solubility‐enhancing tag.

### Design and construction of recombinant plasmids

**Fig. 3:**
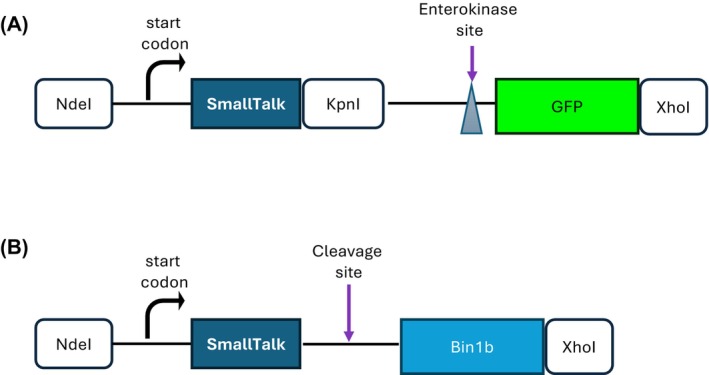
Expression strategy for SmallTalk‐tagged proteins. (A) Gene map of expression cassette for SmallTalk‐GFP. (B) Gene map of expression cassette for SmallTalk‐Bin1b.

Figure 3A shows the construct design for SmallTalk‐GFP. SmallTalk was amplified directly from SmbP template DNA with PCR; NdeI and KpnI restriction sites were introduced at both flanks. The enterokinase cleavage site (DDDDK) is present between SmallTalk and GFP to allow molecular cleavage of SmallTalk to release tag‐free GFP if desired, as done before with the metal‐binding proteins SmbP and CusF3H+ [11, 28].

For the SmallTalk‐Bin1b construct, as per the design shown in Fig. 3B, the coding DNA sequence was synthesized. The amino acid sequence was downloaded from the Antimicrobial Peptide Database (https://aps.unmc.edu/) under the accession number AP01592 and was optimized for codon usage preference in *E. coli*. The synthetic coding DNA sequence was flanked by NdeI and XhoI and was cloned into pET30a(+), as previously done for SmbP‐Bin1b [18].

### Expression and purification of SmallTalk‐tagged proteins

**Fig. 4:**
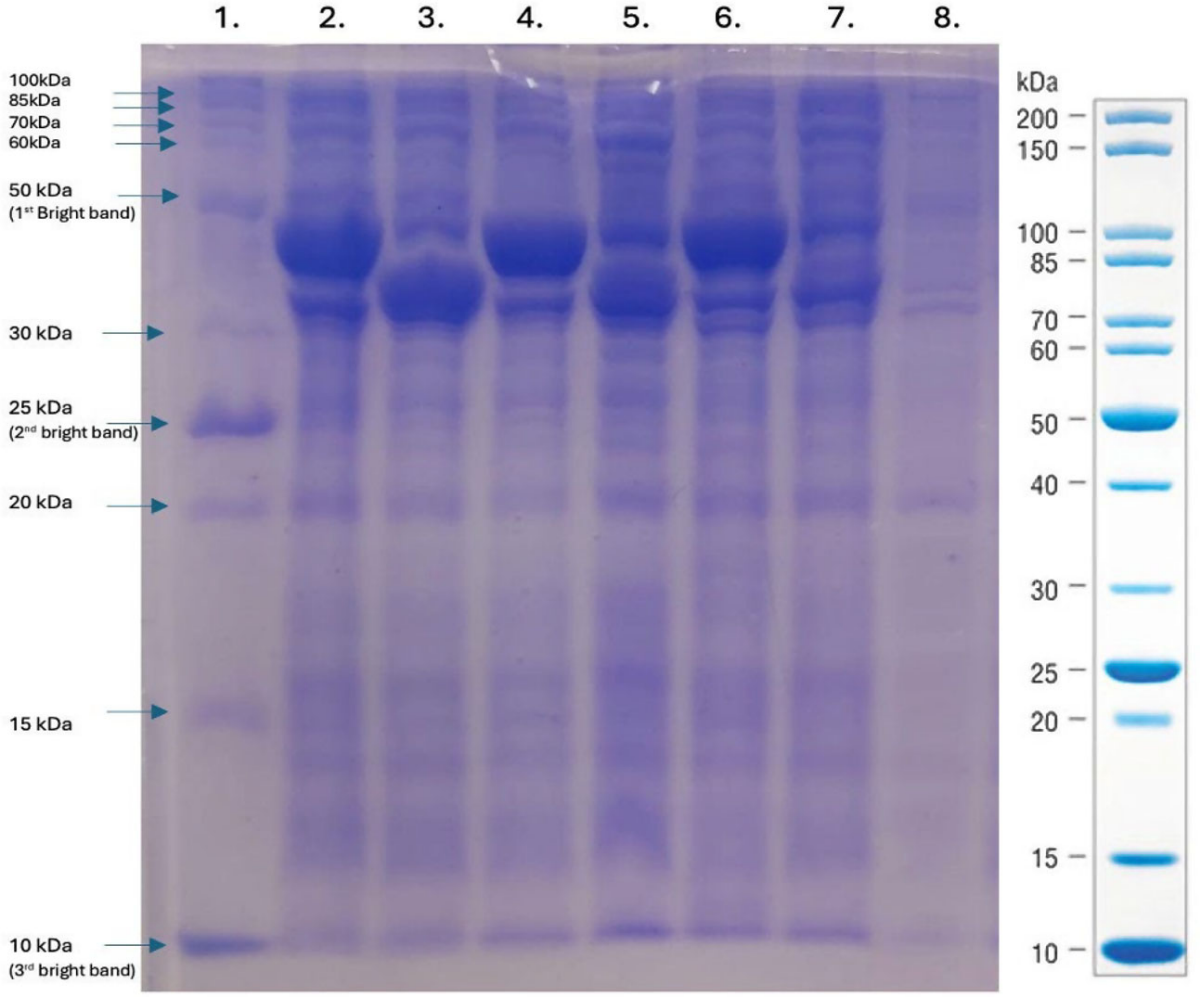
15% SDS/PAGE analysis of small‐scale expression of SmallTalk‐GFP and SmbP‐GFP expressed in *E. coli* BL21(DE3). Lane 1: Unstained protein marker, broad range (10–200 kDa, New England Biolabs); lane 2: SmbP‐GFP (25 °C, 16 h); lane 3: SmallTalk‐GFP (25 °C, 16 h); lane 4: SmbP‐GFP (37 °C, 16 h); lane 5: SmallTalk‐GFP (37 °C, 16 h); lane 6: SmbP‐GFP (37 °C, 4 h); lane 7: SmallTalk‐GFP (37 °C, 4 h); Lane 8: lysate from untransformed cells.

The *E. coli* strain BL21(DE3) was selected for the recombinant production of SmallTalk‐GFP due to its engineered capacity of being protease deficient and producing its own T7 RNA polymerase under the control of the lac promoter. Earlier, the use of SmbP as a carrier protein has shown significant advantages in recombinant expression and purification of the fusion protein in soluble form, including GFP and Bin1b [11, 18]. SmallTalk, a variant of SmbP, is expected to give similar results regarding soluble expression and efficient purification using IMAC. To conduct a comparative analysis of protein expression for SmbP‐GFP and SmallTalk‐GFP, both constructs were expressed at different predefined sets of growth parameters. Cell pellets collected from IPTG‐induced overnight‐grown cell cultures were lysed to obtain the cell lysates with soluble recombinant protein. A 15% SDS/PAGE, shown in Fig. 4, was run to visualize the expression of recombinant GFP tagged with SmallTalk where cell lysates were loaded in the successive wells along with a control. The gel image shows evidence of the expression of SmallTalk‐GFP at comparable levels to the expression of SmbP‐GFP [11]. Results suggest that the conditions of 25 °C and 16 h are the best optimization for the recombinant expression for GFP tagged with SmallTalk.

**Fig. 5:**
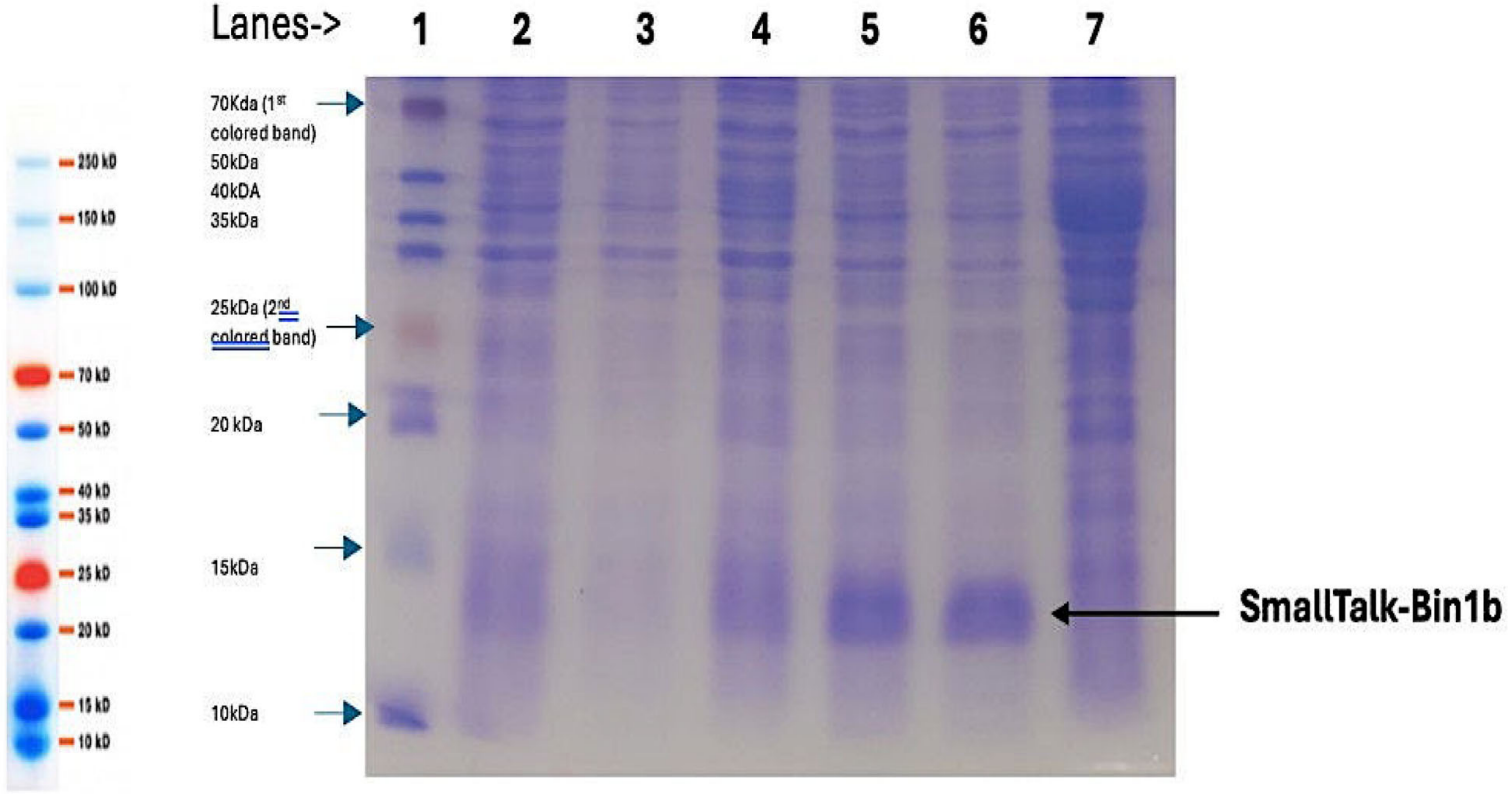
15% SDS/PAGE analysis for the expression of SmallTalk‐Bin1b in *E. coli* SHuffle T7(DE3). Lane 1: BIO BASIC prestained protein marker; lane 2: lysate from uninduced cells (37 °C, 16 h); lane 3: SmallTalk‐Bin1b (37 °C, 4 h); lane 4: SmallTalk‐Bin1b (37 °C, 16 h); lane 5: SmallTalk‐Bin1b (25 °C, 4 h); lane 6: SmallTalk‐Bin1b (25 °C, 16 h); lane 7: lysate from untransformed cells.

For the expression of Bin1b, the SHuffle T7(DE3) strain of *E. coli* was selected since it can produce disulfide bonds in its cytoplasm [18, 29]. The small‐scale expression of SmallTalk‐Bin1b shown in Fig. 5, represents an electrophoretic analysis of clear cell lysates prepared from small‐scale cell cultures under varying sets of growth parameters (time and temperature) and at a constant rotation of 220 r.p.m. Evidence of recombinant protein expression in soluble form at all the growth parameters can be observed, except at 37 °C for 4 h (Fig. 5, lane 3); this can be attributed to the formation of inclusion bodies due to higher temperatures. The most efficient expression was observed at 25 °C for 4 or 16 h (Fig. 5, lanes 5 and 6).

**Fig. 6:**
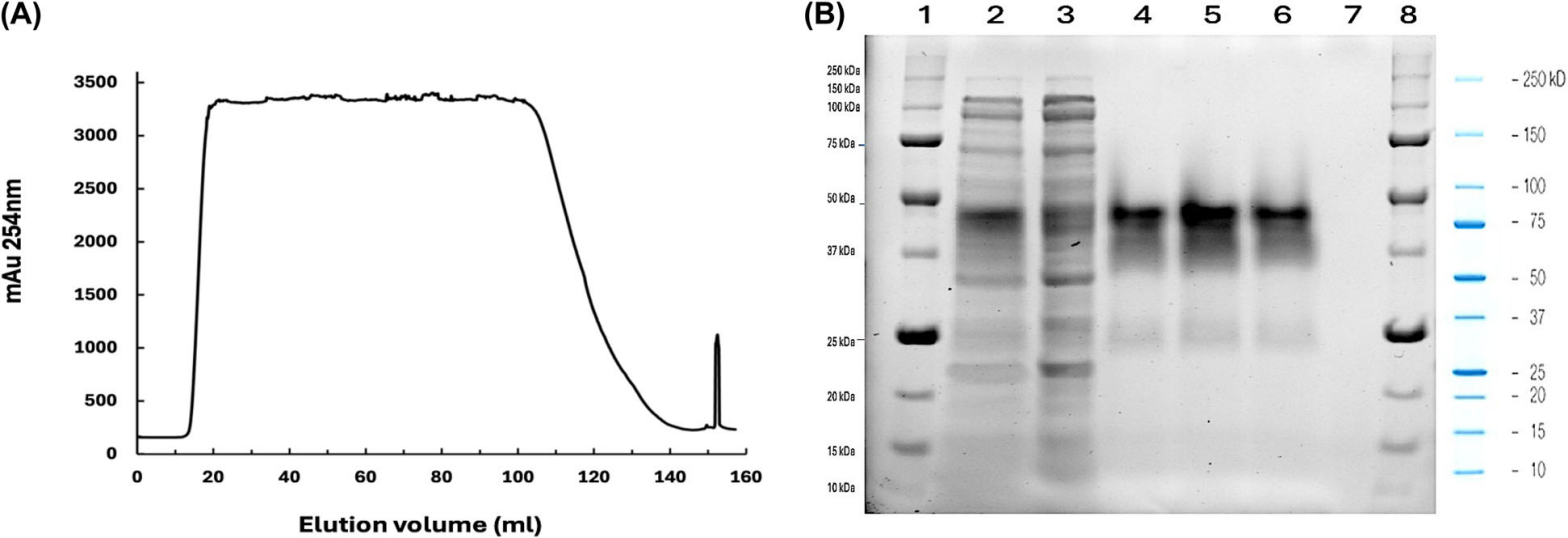
Results from the purification of SmallTalk‐GFP using IMAC chromatography. (A) Chromatogram obtained from the purification of SmallTalk‐GFP using the ÄKTA Prime Plus FPLC system using a 1 mL HisTrap FF column. The column was equilibrated with 50 mm Tris–HCl, 500 mm NaCl, pH 8.0, and washed with 50 mm Tris–HCl, 500 mm NaCl, 2.5 mm imidazole, pH 8.0. One‐step elution was performed using 50 mm Tris–HCl, 500 mm NaCl, and 200 mm imidazole at pH 8.0. The chromatographic elution profile is presented with absorbance at 254 nm plotted on the *Y*‐axis and elution volume (mL) on the *X*‐axis. (B) 12% SDS/PAGE analysis from the purification of SmallTalk‐GFP after IMAC. Lanes 1 and 8: Precision Plus Protein All Blue Prestained protein marker; lane 2: lysate; lane 3: flow‐through; lanes 4 to 6: elution fractions; lane 7: blank.

The ÄKTA Prime Plus HPLC system was used to purify recombinant proteins, deploying the immobilized metal affinity chromatography principle. SmallTalk‐GFP and SmallTalk‐Bin1b were loaded onto a 1‐mL HisTrap FF column charged with Ni(II) ions in separate runs. The hypothesis that SmallTalk could exhibit affinity to nickel ions bound to the resin of the HisTrap FF column, thereby allowing the separation of SmallTalk‐bound proteins from the rest of the cellular proteins present in the cell lysate, was tested at this point. The chromatogram for SmallTalk‐GFP purification is shown in Fig. 6A, with a typical shape of affinity chromatography, where the sharp peak at the end represents the elution using a buffer containing 200 mm imidazole. The bright green color of GFP was visible, representing that the expressed protein is functionally active. The electrophoresis results shown in Fig. 6B demonstrate the presence of SmallTalk‐GFP in the elution fractions, with a purity level of 87%. This indicates the effectiveness of SmallTalk as a fusion tag for metal affinity chromatography. Protein purity was estimated through densitometric analysis using imagej. The gel image was converted to 8‐bit grayscale, and the bands in lanes 4–6 were analyzed with the ‘Gel Analysis’ tool. The area under the curve for each band was quantified, and purity was calculated as the percentage of the main band's intensity relative to the total intensity of all detectable bands in each lane. The absence of SmallTalk‐GFP in the flow‐through fraction, as seen in the SDS/PAGE results, provides evidence of the binding of SmallTalk to the Ni(II) charged agarose resin. Total quantification of SmallTalk‐GFP, determined using the Bradford method, revealed an amount of 7.2 mg from 1 L of cell culture, which is higher than the previously reported yield of SmbP‐GFP at 5.6 mg·L^−1^ [11].

**Fig. 7:**
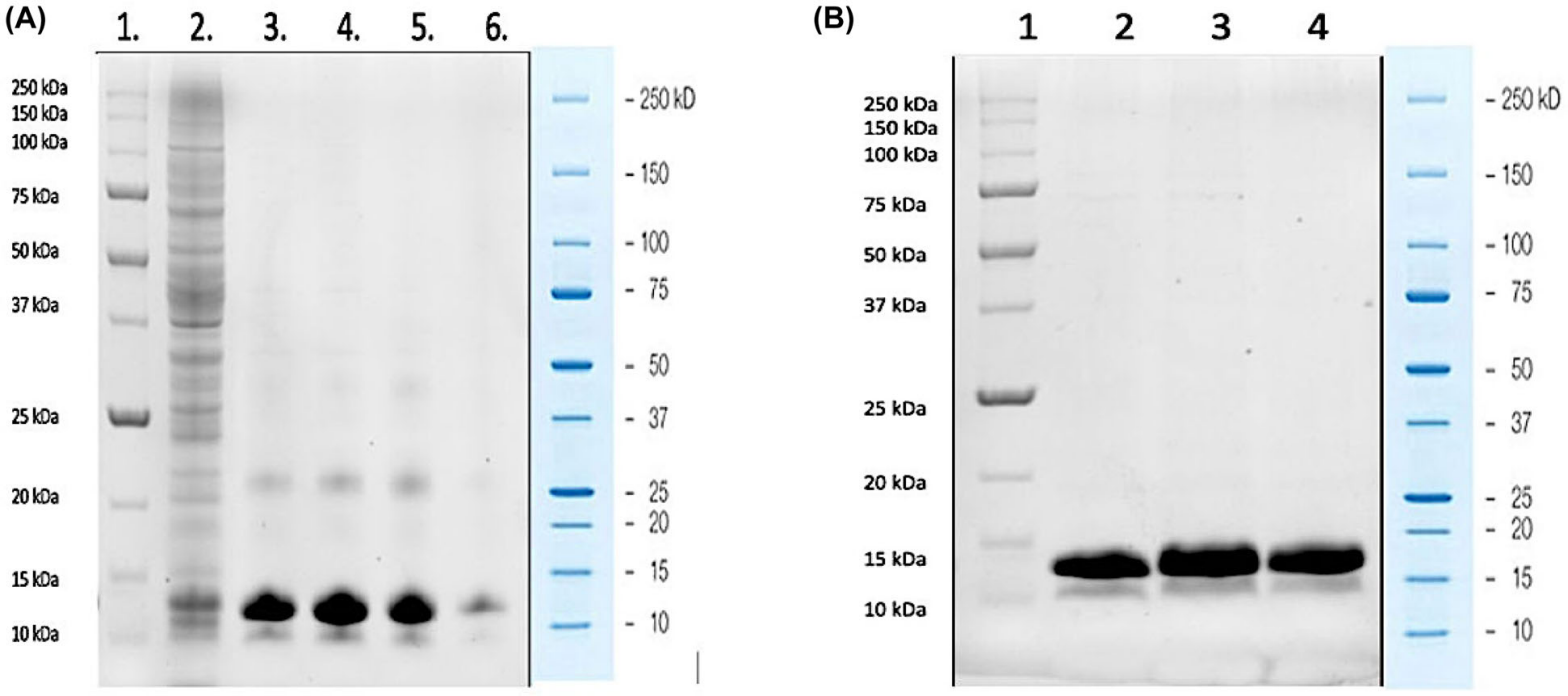
Electrophoretic analysis for the purification of SmallTalk‐Bin1b. (A) A gel was made using the TGX FastCast Acrylamide Kit (12%) and visualized in Bio‐Rad's ChemiDoc MP Imaging System after purification through IMAC. Lane 1: Precision Plus Protein All Blue Prestained protein marker; lane 2: cell lysate; lanes 3–6: elution fractions. (B) SmallTalk‐Bin1b after IMAC and anion exchange chromatography analyzed in TGX FastCast Acrylamide Kit (12%) and visualized in Bio‐Rad's ChemiDoc MP Imaging System. Lane 1: Precision Plus Protein All Blue Prestained protein marker; Lanes 2–4: purified SmallTalk‐Bin1b.

In the case of SmallTalk‐Bin1b, a purity level of 85% was observed after IMAC purification and electrophoretic analysis, as determined by imagej analysis. To assess its antimicrobial activity, further purification was needed through anion exchange chromatography. This additional step allowed the assay to be performed using the complete construct without the need for tag removal or a second round of IMAC. The purification of SmallTalk‐Bin1b was analyzed using SDS/PAGE, and the results are shown in Fig. 7, with a final purity of 90% as determined by imagej analysis. SmallTalk‐Bin1b yield was 6.4 mg per liter of culture under flask‐based expression. The yield of the recombinant Bin1b from a bioreactor‐based 1 L expression was determined using the BCA assay, resulting in an estimated concentration of 9.8 mg·L^−1^. This yield is higher than previously obtained from the flask‐based experiment, which can be attributed to the enhanced control of parameters such as pH, dissolved oxygen, temperature, and agitation in the bioreactor. These optimized conditions likely supported better cell growth and protein expression, resulting in improved overall productivity. A comparison of the yield of Bin1b tagged with different tags is presented in Table 3.

**Table 3:** Comparison of production of Bin1b expressed using multiple fusion proteins.

Fusion protein	Recombinant peptide (mg·L^−1^)	References
GST	2.4	[30]
GB1	5–6	[31]
SmbP	11.0	[18]
CusF3H+	13.6	[18]
SmallTalk (flask‐based expression)	6.4	This study
SmallTalk (Bioreactor‐based expression)	9.8	This study

### Antimicrobial activity of SmallTalk‐Bin1b

The MIC is defined as the lowest concentration of an antimicrobial agent at which no visible bacterial growth is observed; this is determined by visual inspection and confirmed using spectrophotometric analysis, where no change in absorbance indicates the absence of bacterial proliferation. We tested the antimicrobial capacity of SmallTalk‐Bin1b against *Staphylococcus aureus* ATCC 25923, *Escherichia coli* ATCC 25922, and clinical isolates of *Klebsiella pneumoniae* and *Pseudomonas aeruginosa*. The lowest concentration at which no visible growth was observed and quantified in the spectrophotometer was considered the proposed MIC of SmallTalk‐Bin1b. The MIC values against each test pathogen are reported in Table 4. We also determined the MIC values for Bin1b tagged with SmbP against *S. aureus* and *E. coli* for functional comparisons; these are shown in Table 5.

**Table 4:** MIC values of SmallTalk‐Bin1b expressed against Gram‐positive and Gram‐negative bacteria.

Microorganism	MIC (μm)
*Escherichia coli*	20
*Klebsiella pneumoniae*	20
*Pseudomonas aeruginosa*	7.5
*Staphylococcus aureus*	22.5

**Table 5:** MIC values comparison of Bin1b tagged with SmallTalk and SmbP against *E. coli* and *S. aureus*.

Microorganism	MIC (μm) of SmallTalk‐Bin1b	MIC (μm) of SmbP‐Bin1b
*Escherichia coli*	20	25
*Staphylococcus aureus*	22.5	28

The MIC values shown in Tables 4 and 5 represent the average of three technical replicates from a single, representative experiment. This experiment was selected after independent repetitions confirmed its reproducibility. Detailed dose–response plots for each peptide–bacteria combination are provided in Figs S1–S6. These supplemental plots display the individual replicate values, along with the mean and standard deviation, as well as statistical Student's *t*‐test evaluations compared to the untreated negative control. The analysis verifies that the reported MIC values are supported by reproducible experiments, with variability and significance quantified.

SmallTalk‐Bin1b retained total antimicrobial activity against *Staphylococcus aureus*, *Escherichia coli*, *Klebsiella pneumoniae*, and *Pseudomonas aeruginosa*, demonstrating that the SmallTalk tag did not interfere with the functional properties of the peptide. Furthermore, here we report, for the first time, the antimicrobial activity of Bin1b against *K. pneumoniae* and *P. aeruginosa*. SmallTalk‐Bin1b exhibited slightly lower MIC values against *S. aureus* and *E. coli* (22.5 and 20 μm, respectively) than SmbP‐Bin1b (28 and 25 μm). One known mechanism by which beta‐defensins exert their antimicrobial effects is through the aggregation of molecules on bacterial membranes, leading to the formation of pores [32]. The size of fusion proteins can influence this aggregation, potentially hindering their ability to form the necessary structures for effective antimicrobial activity. Previous studies have shown that the recombinant peptide GB1‐Bin1b exhibits antimicrobial properties against *E. coli*, even when fused with a fusion partner. This contrasts with the results observed with the GST‐Bin1b recombinant protein [31, 33]. The size difference between these two fusion proteins is significant: GB1 is a relatively small protein, weighing only 6.4 kDa, while GST has a molecular weight of 26 kDa. In comparison, the SmbP and CusF3H+ proteins weigh around 10 kDa, so it was interesting to observe a behavior like that of GB1. The antimicrobial activity of SmallTalk‐Bin1b demonstrates that SmallTalk is an effective fusion tag for the biologically active peptide Bin1b. This results in lower MIC values compared to SmbP‐Bin1b. The findings indicate consistent activity against both Gram‐positive and Gram‐negative bacterial strains. This suggests that, although SmallTalk yields a lower quantity of protein compared to the metal‐binding proteins CusF3H+ and SmbP, the benefits of using SmallTalk, especially in preserving the bioactivity of Bin1b, outweigh this minor drawback. Overall, the reliability of SmallTalk could provide an effective platform for producing bioactive peptides during the early stages of laboratory research. Additionally, it opens exciting possibilities for future applications in antimicrobial research.

## Conclusions

The SmallTalk tag, although slightly less efficient in terms of total protein yield compared to SmbP, presents distinct advantages in preserving the biological activity of the target protein or peptide. This makes it a promising option for future applications where functional assays are essential and minimizing fusion tag interference is important. For example, in the case of Bin1b, SmallTalk allows for the retention of its antimicrobial activity, which SmbP slightly compromised. These findings underscore the potential of SmallTalk as an effective fusion tag for producing bioactive peptides in a heterologous system, such as *E. coli*.

## Conflict of interest

The authors declare no conflict of interest.

## Author contributions

AT made the DNA constructs, performed the experiments, and wrote the initial manuscript. NGC‐V provided the fully characterized *P. aeruginosa* and *K. pneumoniae* strains from his laboratory at the University Hospital and assisted in evaluating the antimicrobial assays for *S. aureus* and *K. pneumoniae*. AG‐L assisted in the antimicrobial assays for *E. coli* and *P. aeruginosa* and edited the manuscript. XZ designed the experiments, edited the manuscript, and proposed the research as Principal Investigator. All authors read and approved the final manuscript.

## Supporting information


**Fig. S1.** Growth inhibition of *Staphylococcus aureus* by SmallTalk‐Bin1b.
**Fig. S2.** Growth inhibition of *Staphylococcus aureus* by SmbP‐Bin1b.
**Fig. S3.** Growth inhibition of *Escherichia coli* by SmallTalk‐Bin1b.
**Fig. S4.** Growth inhibition of *Escherichia coli* by SmbP‐Bin1b.
**Fig. S5.** Growth inhibition of *Klebsiella pneumoniae* by SmallTalk‐Bin1b.
**Fig. S6.** Growth inhibition of *Pseudomonas aeruginosa* by SmallTalk‐Bin1b.

## Data Availability

The data supporting the findings of this study are available from the corresponding author upon request.

## References

[1] Rosano GL and Ceccarelli EA (2014) Recombinant protein expression in *Escherichia coli*: advances and challenges. Front Microbiol 5, 172. 24860555 10.3389/fmicb.2014.00172 PMC4029002

[2] Ki MR and Pack SP (2020) Fusion tags to enhance heterologous protein expression. Appl Microbiol Biotechnol 104, 2411–2425. 31993706 10.1007/s00253-020-10402-8

[3] Oliveira C and Domingues L (2018) Guidelines to reach high‐quality purified recombinant proteins. Appl Microbiol Biotechnol 102, 81–92. 29151158 10.1007/s00253-017-8623-8

[4] Costa S , Almeida A , Castro A and Domingues L (2014) Fusion tags for protein solubility, purification, and immunogenicity in *Escherichia coli*: the novel Fh8 system. Front Microbiol 5, 63. 24600443 10.3389/fmicb.2014.00063 PMC3928792

[5] Smith DB and Johnson KS (1988) Single‐step purification of polypeptides expressed in *Escherichia coli* as fusions with glutathione S‐transferase. Gene 67, 31–40. 3047011 10.1016/0378-1119(88)90005-4

[6] di Guan C , Li P , Riggs PD and Inouye H (1988) Vectors that facilitate the expression and purification of foreign peptides in *Escherichia coli* by fusion to maltose‐binding protein. Gene 67, 21–30. 2843437 10.1016/0378-1119(88)90004-2

[7] Muttenthaler M , King GF , Adams DJ and Alewood PF (2021) Trends in peptide drug discovery. Nat Rev Drug Discov 20, 309–325. 33536635 10.1038/s41573-020-00135-8

[8] Esposito D and Chatterjee DK (2006) Enhancement of soluble protein expression through the use of fusion tags. Curr Opin Biotechnol 17, 353–358. 16781139 10.1016/j.copbio.2006.06.003

[9] Mahmoudi Gomari M , Saraygord‐Afshari N , Farsimadan M , Rostami N , Aghamiri S and Farajollahi MM (2020) Opportunities and challenges of the tag‐assisted protein purification techniques: applications in the pharmaceutical industry. Biotechnol Adv 45, 107653. 33157154 10.1016/j.biotechadv.2020.107653

[10] Barney BM , LoBrutto R and Francisco WA (2004) Characterization of a small metal binding protein from *Nitrosomonas europaea* . Biochemistry 43, 11206–11213. 15366930 10.1021/bi049318k

[11] Vargas‐Cortez T , Morones‐Ramirez JR , Balderas‐Renteria I and Zarate X (2015) Expression and purification of recombinant proteins in *Escherichia coli* tagged with a small metal‐binding protein from *Nitrosomonas europaea* . Protein Expr Purif 118, 49–54. 26494603 10.1016/j.pep.2015.10.009

[12] Gomez‐Lugo JJ , Santos BD , Perez‐Perez DA , Montfort‐Gardeazabal JM , McEvoy MM and Zarate X (2021) Expression and purification of recombinant proteins in *Escherichia coli* tagged with the metal‐binding proteins SmbP and CusF3H+. In Protein Downstream Processing: Design, Development, and Application of High and Low‐Resolution Methods ( Labrou NE , ed.), pp. 329–344. Springer US, New York, NY. 10.1007/978-1-0716-0775-6_22 33128759

[13] Perez‐Perez DA , Pioquinto‐Avila E , Arredondo‐Espinoza E , Morones‐Ramirez JR , Balderas‐Renteria I and Zarate X (2020) Engineered small metal‐binding protein tag improves the production of recombinant human growth hormone in the periplasm of *Escherichia coli* . FEBS Open bio 10, 546–551. 10.1002/2211-5463.12808 PMC7137794 32049439

[14] Perez‐Perez DA , Villanueva‐Ramirez T d J , Hernandez‐Pedraza AE , Casillas‐Vega NG , Gonzalez‐Barranco P and Zarate X (2021) The small metal‐binding protein SmbP simplifies the recombinant expression and purification of the antimicrobial peptide LL‐37. Antibiotics 10, 1271. 34680851 10.3390/antibiotics10101271 PMC8532731

[15] Narh JK , Casillas‐Vega NG and Zarate X (2024) LL‐37_Renalexin hybrid peptide exhibits antimicrobial activity at lower MICs than its counterpart single peptides. Appl Microbiol Biotechnol 108, 126. 38229302 10.1007/s00253-023-12887-5 PMC10787891

[16] Gomez‐Lugo JJ , Casillas‐Vega NG , Gomez‐Loredo A , Balderas‐Renteria I and Zarate X (2024) High‐yield expression and purification of Scygonadin, an antimicrobial peptide, using the small metal‐binding protein SmbP. Microorganisms 12, 278. 38399682 10.3390/microorganisms12020278 PMC10893511

[17] Montfort‐Gardeazabal JM , Claudio PCM‐S , Casillas‐Vega NG and Zarate X (2020) Expression and purification of the VpDef Defensin in *Escherichia coli* using the small metal‐binding proteins CusF3H+ and SmbP. Protein Pept Lett 28, 108–114. 10.2174/0929866527666200610133407 32520670

[18] Montfort‐Gardeazabal JM , Balderas‐Renteria I , Casillas‐Vega NG and Zarate X (2021) Expression and purification of the antimicrobial peptide Bin1b in *Escherichia coli* tagged with the fusion proteins CusF3H+ and SmbP. Protein Expr Purif 178, 105784. 33129981 10.1016/j.pep.2020.105784

[19] Magana M , Pushpanathan M , Santos AL , Leanse L , Fernandez M , Ioannidis A , Giulianotti MA , Apidianakis Y , Bradfute S , Ferguson AL *et al*. (2020) The value of antimicrobial peptides in the age of resistance. Lancet Infect Dis 20, e216–e230. 32653070 10.1016/S1473-3099(20)30327-3

[20] Aljeldah MM (2022) Antimicrobial resistance and its spread is a global threat. Antibiotics 11, 1082. 36009948 10.3390/antibiotics11081082 PMC9405321

[21] Hartig SM (2013) Basic image analysis and manipulation in ImageJ. Curr Protoc Mol Biol 102, 14.15.1–14.15.12. 10.1002/0471142727.mb1415s102 23547012

[22] Schneider CA , Rasband WS and Eliceiri KW (2012) NIH image to ImageJ: 25 years of image analysis. Nat Methods 9, 671–675. 22930834 10.1038/nmeth.2089 PMC5554542

[23] Kowalska‐Krochmal B and Dudek‐Wicher R (2021) The minimum inhibitory concentration of antibiotics: methods, interpretation, clinical relevance. Pathogens 10, 165. 33557078 10.3390/pathogens10020165 PMC7913839

[24] Hameduh T , Haddad Y , Adam V and Heger Z (2020) Homology modeling in the time of collective and artificial intelligence. Comput Struct Biotechnol J 18, 3494–3506. 33304450 10.1016/j.csbj.2020.11.007 PMC7695898

[25] Kumar R and Sharma A (2023) Computational strategies and tools for protein tertiary structure prediction. In Basic Biotechniques for Bioprocess and Bioentrepreneurshippp. 225–242. Academic Press, London.

[26] Hollingsworth SA and Karplus PA (2010) A fresh look at the Ramachandran plot and the occurrence of standard structures in proteins. Biomol Concepts 1, 271–283. 21436958 10.1515/BMC.2010.022 PMC3061398

[27] Chen VB , Arendall WB , Headd JJ , Keedy DA , Immormino RM , Kapral GJ , Murray LW , Richardson JS and Richardson DC (2010) MolProbity: all‐atom structure validation for macromolecular crystallography. Acta Crystallogr D Biol Crystallogr 66, 12–21. 20057044 10.1107/S0907444909042073 PMC2803126

[28] Vargas‐Cortez T , Morones‐Ramirez JR , Balderas‐Renteria I and Zarate X (2017) Production of recombinant proteins in *Escherichia coli* tagged with the fusion protein CusF3H+. Protein Expr Purif 132, 44–49. 28087367 10.1016/j.pep.2017.01.006

[29] Lobstein J , Emrich CA , Jeans C , Faulkner M , Riggs P and Berkmen M (2012) SHuffle, a novel *Escherichia coli* protein expression strain capable of correctly folding disulfide bonded proteins in its cytoplasm. Microb Cell Fact 11, 753. 10.1186/1475-2859-11-56 PMC3526497 22569138

[30] Sun XJ , Wang DN , Zhang WJ and Wu XF (2004) Expression of an antimicrobial peptide identified in the male reproductive system of rats. Mol Biotechnol 28, 185–189. 15542918 10.1385/MB:28:3:185

[31] Guo C , Diao H , Lian Y , Yu H , Gao H , Zhang Y and Lin D (2009) Recombinant expression and characterization of an epididymis‐specific antimicrobial peptide BIN1b/SPAG11E. J Biotechnol 139, 33–37. 19007826 10.1016/j.jbiotec.2008.10.003

[32] Li Y (2011) Recombinant production of antimicrobial peptides in *Escherichia coli*: a review. Protein Expr Purif 80, 260–267. 21843642 10.1016/j.pep.2011.08.001

[33] Li P , Chan HC , He B , So SC , Chung YW , Shang Q , Zhang YD and Zhang YL (2001) An antimicrobial peptide gene found in the male reproductive system of rats. Science 291, 1783–1785. 11230693 10.1126/science.1056545

