# Supplementary material for: SmallTalk: a novel small‐sized fusion tag for peptide expression and purification
Source: FEBS Open Bio. 2025 Nov 11;16(3):461–73. doi: 10.1002/2211-5463.70147 (PMC12955753; doi:10.1002/2211-5463.70147)
Supplement: Supplementary file 1 — Fig. S1. Growth inhibition of Staphylococcus aureus by SmallTalk‐Bin1b. Fig. S2. Growth inhibition of Staphylococcus aureus by SmbP‐Bin1b. Fig. S3. Growth inhibition of Escherichia coli by SmallTalk‐Bin1b. Fig. S4. Growth inhibition of Escherichia coli by SmbP‐Bin1b. Fig. S5. Growth inhibition of Klebsiella pneumoniae by SmallTalk‐Bin1b. Fig. S6. Growth inhibition of Pseudomonas aeruginosa by SmallTalk‐Bin1b. [file FEB4-16-461-s001.zip › feb470147-sup-0001-Figures.pdf]

SmallTalk: A novel small-sized fusion tag for peptide expression and purification

Atika Tariq, Nestor G. Casillas-Vega, Alma Gomez-Loredo, and Xristo Zarate

Antimicrobial assays with fusion proteins SmallTalk-Bin1b and SmbP-Bin1b

Bacterial growth was measured by optical density (OD) at 600 nm after a 16-hour incubation period with varying concentrations of peptides. Each treatment included three independent biological replicates (n = 3). To correct for baseline differences, pre-incubation absorbance values were subtracted from post-incubation readings. The data were then normalized to the untreated control (1X PBS), which was set at 100% growth. Results are presented as the mean ± standard deviation. Statistical significance relative to the untreated control was evaluated using Student's t-tests. Significance levels are indicated as follows: $p < 0.05$ (*), $p < 0.01$ (**), and $p < 0.001$ (***).

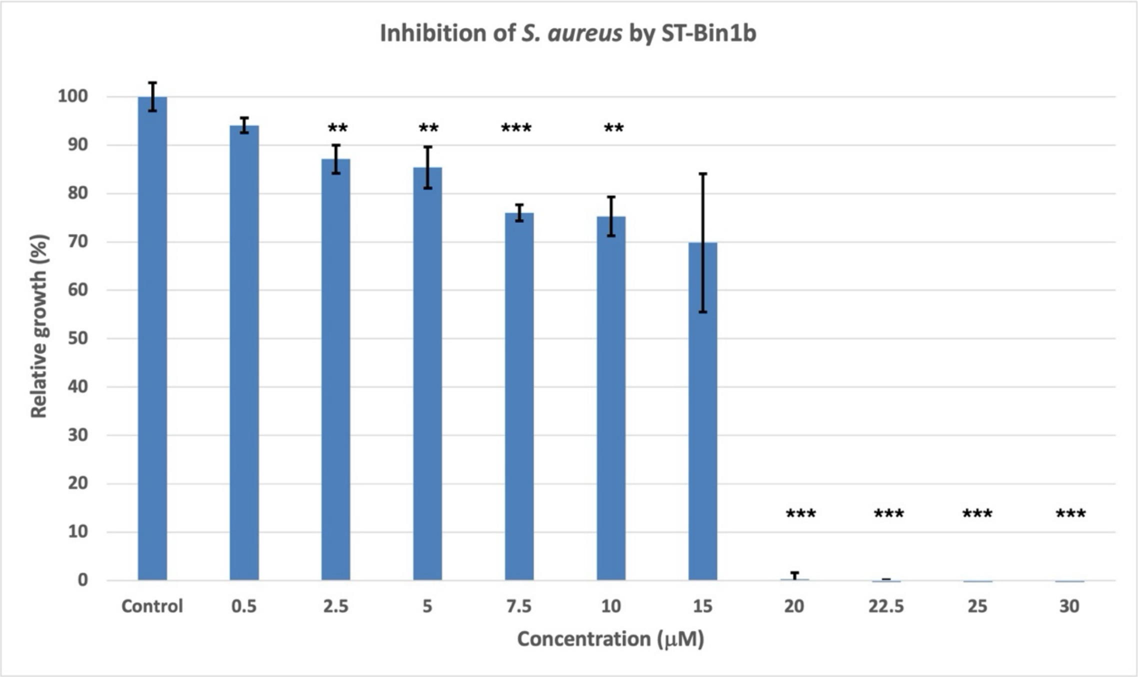

Figure S1. Growth inhibition of *Staphylococcus aureus* by SmallTalk-Bin1b. The antibacterial activity of SmallTalk-Bin1b was assessed against *S. aureus* by measuring OD_{600} after 16 h of incubation. Values represent mean ± SD. Statistical significance relative to the control (1X PBS) was determined using Student's *t*-test; $p < 0.01$ (**) and $p < 0.001$ (***).

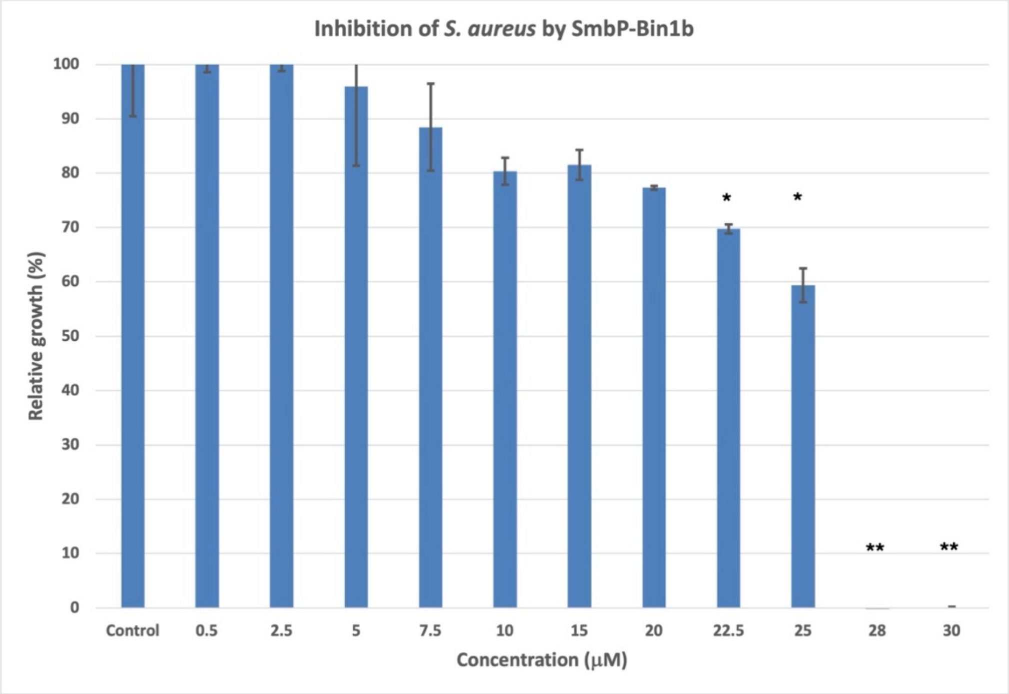

Figure S2. Growth inhibition of *Staphylococcus aureus* by SmbP-Bin1b. The antibacterial activity of SmbP-Bin1b was assessed against *S. aureus* by measuring OD_{600} after 16 h of incubation. Values represent mean ± SD. Statistical significance relative to the control (1X PBS) was determined using Student's *t*-test; $p < 0.05$ (*) and $p < 0.01$ (**).

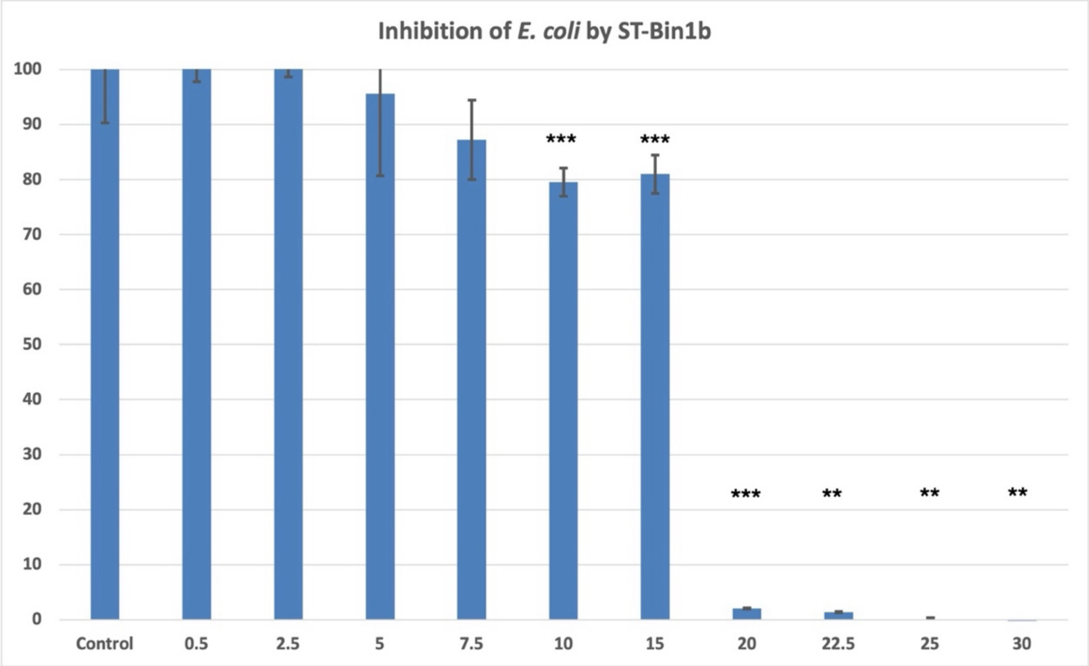

Figure S3. Growth inhibition of *Escherichia coli* by SmallTalk-Bin1b. The antibacterial activity of SmallTalk-Bin1b was assessed against *E. coli* by measuring OD_{600} after 16 h of incubation. Values represent mean ± SD. Statistical significance relative to the control (1X PBS) was determined using Student's *t*-test; $p < 0.01$ (**) and $p < 0.001$ (***).

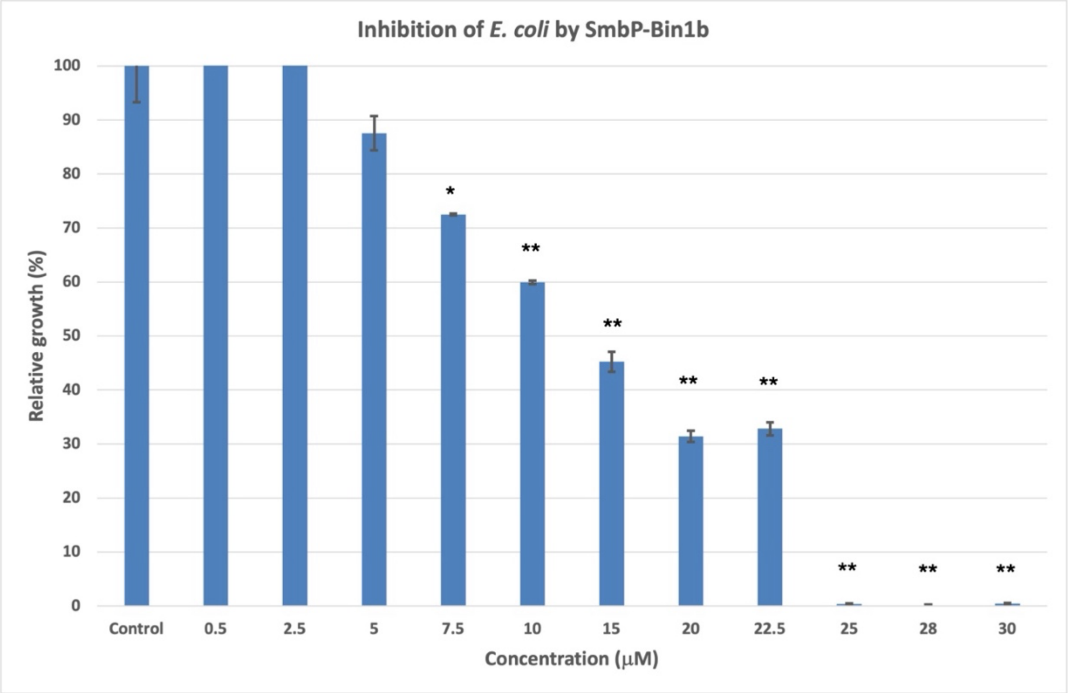

Figure S4. Growth inhibition of *Escherichia coli* by SmbP-Bin1b. The antibacterial activity of SmbP-Bin1b was assessed against *E. coli* by measuring OD_{600} after 16 h of incubation. Values represent mean ± SD. Statistical significance relative to the control (1X PBS) was determined using Student's *t*-test; $p < 0.05$ (*) and $p < 0.01$ (**).

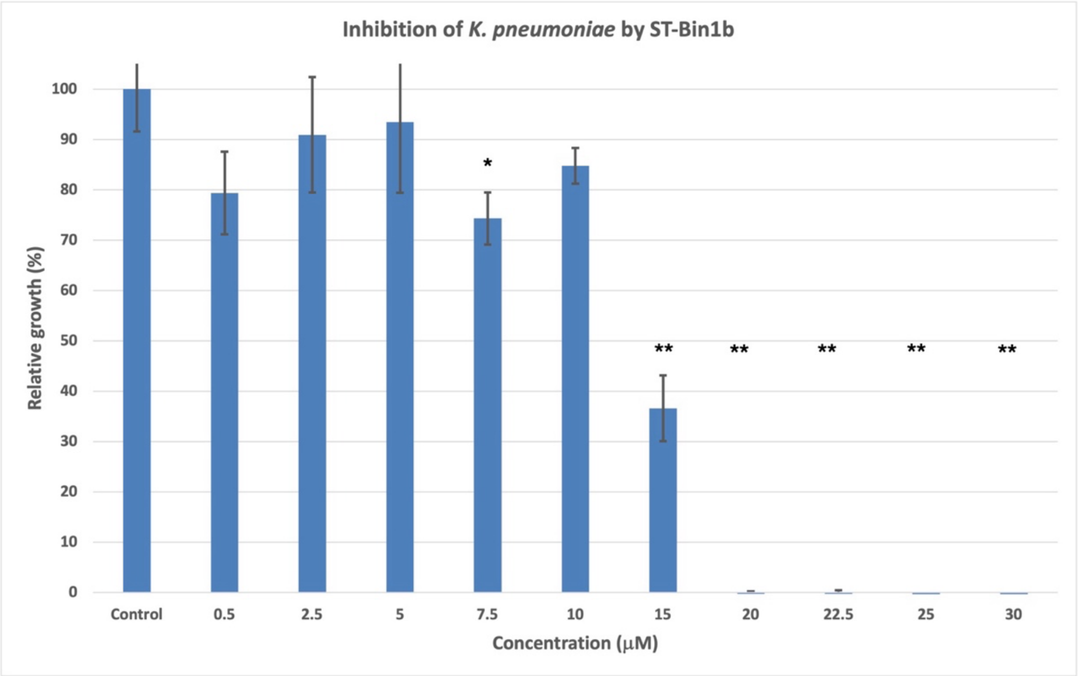

Figure S5. Growth inhibition of *Klebsiella pneumoniae* by SmallTalk-Bin1b. The antibacterial activity of SmallTalk-Bin1b was assessed against *K. pneumoniae* by measuring OD_{600} after 16 h of incubation. Values represent mean ± SD. Statistical significance relative to the control (1X PBS) was determined using Student's *t*-test; $p < 0.05$ (*) and $p < 0.01$ (**).

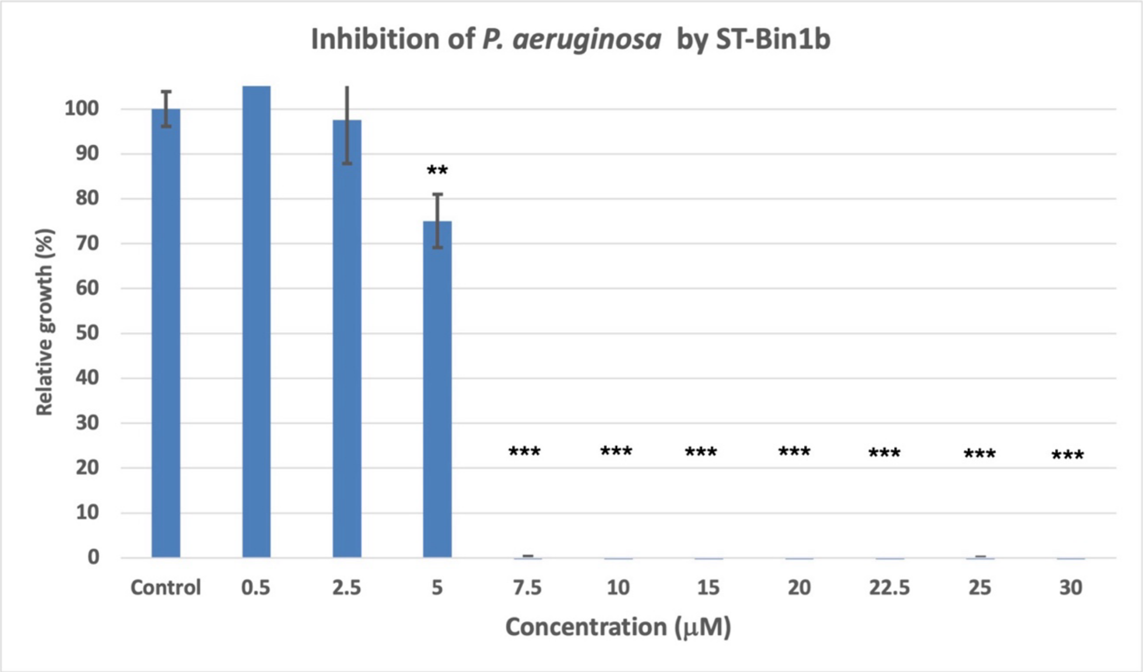

Figure S6. Growth inhibition of *Pseudomonas aeruginosa* by SmallTalk-Bin1b. The antibacterial activity of SmallTalk-Bin1b was assessed against *P. aeruginosa* by measuring OD_{600} after 16 h of incubation. Values represent mean ± SD. Statistical significance relative to the control (1X PBS) was determined using Student's *t*-test; $p < 0.01$ (**) and $p < 0.001$ (***).
