# Supplementary material for: SmallTalk: a novel small‐sized fusion tag for peptide expression and purification
Source: FEBS Open Bio. 2025 Nov 11;16(3):461–73. doi: 10.1002/2211-5463.70147 (PMC12955753; doi:10.1002/2211-5463.70147)
Supplement: Supplementary file 1 — Fig. S1. Growth inhibition of Staphylococcus aureus by SmallTalk‐Bin1b. Fig. S2. Growth inhibition of Staphylococcus aureus by SmbP‐Bin1b. Fig. S3. Growth inhibition of Escherichia coli by SmallTalk‐Bin1b. Fig. S4. Growth inhibition of Escherichia coli by SmbP‐Bin1b. Fig. S5. Growth inhibition of Klebsiella pneumoniae by SmallTalk‐Bin1b. Fig. S6. Growth inhibition of Pseudomonas aeruginosa by SmallTalk‐Bin1b. [file FEB4-16-461-s001.zip › Supporting information.docx]

Supporting information

Figures S1–S6 illustrate the antimicrobial activity assays for the recombinant constructs SmallTalk-Bin1b and SmbP-Bin1b against various bacterial strains. The plots display the normalized bacterial growth (mean ± SD, n = 3) at increasing concentrations of the peptide, relative to the untreated control (1X PBS, 100% growth). Statistical analyses were conducted using Student's t-tests to identify significant differences compared to the control. These results support the MIC determinations presented in Tables 4 and 5 of the manuscript, confirming the reproducibility and statistical validity of the experiments.
